# Intersectional and Marginal Debiasing in Prediction Models for Emergency Admissions

**DOI:** 10.1001/jamanetworkopen.2025.12947

**Published:** 2025-05-29

**Authors:** Elle Lett, Shakiba Shahbandegan, Yuval Barak-Corren, Andrew M. Fine, William G. La Cava

**Affiliations:** 1 Center for Anti-Racism and Community Health, University of Washington School of Public Health, Seattle; 2 Health Systems and Population Health, University of Washington School of Public Health, Seattle; 3 Computational Health Informatics Program, Boston Children’s Hospital, Boston, Massachusetts; 4 Department of Computer Science and Engineering, Michigan State University, East Lansing; 5 Division of Cardiology, The Children’s Hospital of Philadelphia, Philadelphia, Pennsylvania; 6 Division of Emergency Medicine, Boston Children’s Hospital, Boston, Massachusetts; 7 Department of Pediatrics, Harvard Medical School, Boston, Massachusetts

## Abstract

**Importance:**

Fair clinical prediction models are crucial for achieving equitable health outcomes. Intersectionality has been applied to develop algorithms that address discrimination among intersections of protected attributes (eg, Black women rather than Black persons or women separately), yet most fair algorithms default to marginal debiasing, optimizing performance across simplified patient subgroups.

**Objective:**

To assess the extent to which simplifying patient subgroups during training is associated with intersectional subgroup performance in emergency department (ED) admission models.

**Design, Setting, and Participants:**

This prognostic study of admission prediction models used retrospective data from ED visits to Beth Israel Deaconess Medical Center Medical Information Mart for Intensive Care IV (MIMIC-IV; n = 160 016) from January 1, 2011, to December 31, 2019, and Boston Children’s Hospital (BCH; n = 22 222) from June 1 through August 13, 2019. Statistical analysis was conducted from January 2022 to August 2024.

**Main Outcomes and Measures:**

The primary outcome was admission to an in-patient service. The accuracy of admission predictions among intersectional subgroups was measured under variations on model training with respect to optimizing for group level performance. Under different fairness definitions (calibration, error rate balance) and modeling methods (linear, nonlinear), overall performance and subgroup performance of marginal debiasing approaches were compared with intersectional debiasing approaches. Subgroups were defined by self-reported race and ethnicity and gender. Measures include area under the receiver operator characteristic curve (AUROC), area under the precision recall curve, subgroup calibration error, and false-negative rates.

**Results:**

The MIMIC-IV cohort included 160 016 visits (mean [SD] age, 53.0 [19.3] years; 57.4% female patients; 0.3% American Indian or Alaska Native patients, 3.7% Asian patients, 26.2% Black patients, 10.0% Hispanic or Latino patients, and 59.7% White patients; 29.5% admitted) and the BCH cohort included 22 222 visits (mean [SD] age, 8.2 [6.8] years; 52.1% male patients; 0.1% American Indian or Alaska Native patients, 4.0% Asian patients, 19.7% Black patients, 30.6% Hispanic or Latino patients, 0.2% Native Hawaiian or Pacific Islander patients, 37.7% White patients; 16.3% admitted). Among MIMIC-IV groups, intersectional debiasing was associated with a reduced subgroup calibration error from 0.083 to 0.065 (22.3%), while marginal fairness debiasing was associated with a reduced subgroup calibration error from 0.083 to 0.074 (11.3%; difference, 11.1%); among BCH groups, intersectional debiasing was associated with a reduced subgroup calibration error from 0.111 to 0.080 (28.3%), while marginal fairness debiasing was associated with a reduced subgroup calibration error from 0.111 to 0.086 (22.6%; difference, 5.7%). Among MIMIC-IV groups, intersectional debiasing was associated with lowered subgroup false-negative rates from 0.142 to 0.125 (11.9%), while marginal debiasing was associated with lowered subgroup false-negative rates from 0.142 to 0.132 (6.8%; difference, 5.1%). Fairness improvements did not decrease overall accuracy compared with baseline models (eg, MIMIC-IV: mean [SD] AUROC, 0.85 [0.00], both models). Intersectional debiasing was associated with lowered error rates in several intersectional subpopulations compared with other strategies.

**Conclusions and Relevance:**

This study suggests that intersectional debiasing better mitigates performance disparities across intersecting groups than marginal debiasing for admission prediction. Intersectionally debiased models were associated with reduced group-specific errors without compromising overall accuracy. Clinical risk prediction models should consider incorporating intersectional debiasing into their development.

## Key Points

**Question:**

How important is it to explicitly optimize for equity among intersectional, rather than marginal, patient groups when using fair machine learning approaches to develop emergency admission prediction models?

**Findings:**

In a prognostic study using retrospective data from 2 emergency departments and several modeling approaches, intersectional debiasing approaches were associated with reduced subgroup calibration errors by an additional 5.7% to 11.1% and subgroup false-negative rates by an additional 4.5% compared with marginal debiasing.

**Meaning:**

These findings highlight the importance of considering the interacting aspects of patient identity in model development and suggest that intersectional debiasing would be a promising criterion standard for ensuring equity in clinical prediction models.

## Teaser

This prognostic study assesses the extent to which optimization of algorithmic fairness on marginal patient subgroups during training is associated with intersectional, vs marginal, subgroup performance in emergency department (ED) admission models.

## Introduction

Emergency departments (EDs) are dynamic environments where patients present with varying acuity, requiring tailored and efficient treatment plans that prioritize achieving desired health outcomes while optimizing clinician workflow and hospital resource utilization. Emergency departments often function as safety-net care for marginalized populations with reduced economic resources or health care access, particularly among members of ethnoracial minority groups in the US.^1^ These populations also experience the most severe health inequities in disease burden, mortality, and morbidity.^2,3,4^ Racialized health inequities also manifest throughout the ED workflow; Black and Hispanic or Latino patients are subject to longer wait times for initial evaluation by a physician in the ED,^5^ despite data suggesting that Black and Hispanic individuals account for a disproportionate amount of ED visits and are more likely to be repeat visitors.^1^ After initial triage, Black patients who are designated for admission also experience longer ED boarding times,^6^ with such delays associated with adverse health outcomes (including intensive care unit mortality rates^7^ and ventilator-associated pneumonia).^8^

### Machine Learning Models’ Capacity for Improving ED Patient Management

A key challenge in ED workflow is coordinating admissions for patients needing inpatient care, as hospital beds are a limited resource. Bed coordination—the assignment of patients to care teams and beds—can create bottlenecks, increasing ED boarding times and delaying treatment when demand exceeds capacity or allocation is inefficient. Machine learning (ML) models can help accelerate this process by identifying potential admissions early during triage and initial workup, before the formal decision to admit is made (eFigure 1 in Supplement 1). Our study builds on previous ED admission prediction models that have shown strong performance in adult^9^ and pediatric^10^ settings, improving and complementing the assessments of patient disposition made by attending physicians.^11^

### Fairness and Intersectionality

Previous ED admission models optimize overall performance without addressing differences in subgroup outcomes. Given existing inequities in ED wait and board times, a “fairness-agnostic” model could narrow, maintain, or even widen disparities. Therefore, we have developed “fairness-aware” models that optimize both overall accuracy and performance across groups defined by demographic traits. Prior work has noted that many fairness approaches limit their focus to groups defined by a single demographic trait, such as race and ethnicity, or consider multiple demographic traits in isolation (ie, race and ethnicity and gender separately).^12^ We refer to these approaches as “marginal,” as they focus on the marginal distribution of protected attributes while ignoring groups defined by their intersections. Marginal fairness approaches are subject to “fairness gerrymandering,”^13,14^ wherein models that are considered fair for groups defined by single attributes (ie, Black people separately or women separately) still exhibit unfair performance for groups defined by intersections of protected attributes (ie, Native American women or Latino men). We provide a breakdown of currently available, fair ML algorithms and their support for intersecting subgroup definitions in eTable 1 in Supplement 1.

Approaches to mitigate fairness gerrymandering are rooted in intersectionality, a framework established by Crenshaw^15,16^ and Collins,^17^ with origins in social movements in the 1830s.^18,19,20^ Intersectionality views systems of oppression, such as racism and cissexism, as co-occurring, emphasizing that analyzing a single axis of discrimination—such as race and ethnicity—fails to capture the harms experienced by individuals facing multiple forms of discrimination.^21^ Previous work shows how this framework applies to ML fairness throughout different stages of the prediction task, from defining to evaluating and updating the task.^12^

In algorithmic fairness, this framework motivates intersectional fairness that constrains model performance across groups that are defined by the intersections of protected attributes, rather than marginal fairness that is concerned only with the groups defined by the marginal distributions of 1 or more protected attributes. Theoretically, intersectional fairness is ideal; in practice, it can be difficult to achieve due to scarce data on multiply marginalized groups.

### Debiasing and Evaluating Fairness

Fairness metrics must be chosen carefully based on the context of the application.^12^ Depending on the hospital’s patient population, ED traffic, and operating practices, different metrics may best optimize care across groups. For example, in an ED with particularly high ethnoracial inequities in boarding wait times, ensuring fair calibration would ensure that specific groups are not deprioritized or overprioritized via assigned risk scores. Ensuring low subgroup false-negative rates (FNRs), meanwhile, would help ensure that no single group is falsely discharged at a higher rate. To cover the breadth of potential use case scenarios, we focus on 2 fundamental notions of fairness: sufficiency (ie, patients with the same risk score should experience outcomes at a rate that is independent of group membership) and separation (ie, patients with the same outcomes should receive risk scores that are independent of group membership).^22^ For example, if an ED admission model meets sufficiency, patients with a 90% risk score should have equal likelihoods of admission regardless of group membership. Conversely, if the model meets separation, risk scores for admitted patients should not differ by group, meaning FNRs and false-positive rates (FPRs) should be the same across groups. Both traits are important characteristics for fair prediction models, yet cannot be simultaneously satisfied when admission rates differ among groups.^23^ Hence, we study 2 fairness algorithms: one that achieves sufficiency by optimizing group-level calibration, and one that achieves a relaxation of separation by minimizing group-level FNRs. The first algorithm, multicalibration boosting,^24^ is a postprocessing algorithm that constrains the group-level calibration error. The second, fairness-oriented multiobjective optimization (FOMO),^25^ is a training algorithm that we use to constrain group-level FNRs.

In our experiments, we evaluated the ED admission prediction task (eFigure 1 in Supplement 1) across adult and pediatric populations in 2 Boston-based health care centers (Table). We compared the performance of marginal and intersectional debiasing with multicalibration and FOMO, specifically with (1) no debiasing, (2) marginal debiasing based on single attributes (ethnoracial group or gender) or multiple attributes concomitantly (ethnoracial group and gender), and (3) intersectional debiasing based on ethnoracial group and gender. We implemented these debiasing approaches on both logistic regression and random forest models. The overall goal of the study is to measure the extent to which optimization of algorithmic fairness on marginal groups transfers to intersectional patient groups, under different definitions of fairness, models, and clinical settings.

**Table.** Patient Visit Characteristics for the MIMIC-IV and BCH Data

Characteristic	Overall, No. (%)	Discharged, No. (%)	Admitted, No. (%)
**MIMIC-IV ED, 2011-2019**			
Visits	160 016 (100)	112 733 (70.5)	47 283 (29.5)
Patients, No.	90 005	51 306	38 699
Age at visit, mean (SD), y	53.0 (19.3)	49.4 (18.5)	61.6 (18.4)
ED triage acuity^a^			
1	8720 (5.5)	2530 (2.3)	6190 (13.7)
2	47 570 (30.2)	24743 (22.1)	22 827 (50.4)
3	90 948 (57.7)	74 755 (66.6)	16 193 (35.7)
4	9922 (6.3)	9809 (8.7)	113 (0.2)
5	382 (0.2)	376 (0.3)	6 (0.01)
Ethnoracial group			
American Indian or Alaska Native	427 (0.3)	275 (0.2)	152 (0.3)
Asian	5979 (3.7)	3904 (3.5)	2075 (4.4)
Black or African American	41 944 (26.2)	36 217 (32.1)	5727 (12.1)
Hispanic or Latino	16 057 (10.0)	13 826 (12.3)	2231 (4.7)
White	95 609 (59.7)	58 511 (51.9)	37 098 (78.5)
Gender			
Women	91 774 (57.4)	68 327 (60.6)	23 447 (49.6)
Men	68 242 (42.6)	44 406 (39.4)	23 836 (50.4)
**BCH ED, 2019**			
Visits	22 222 (100)	18 605 (83.7)	3617 (16.3)
Patients, No.^b^	17 938	15 533	3069
Age at visit, mean (SD), y	8.2 (6.8)	8.0 (6.6)	9.5 (7.6)
ED triage acuity			
1	130 (0.6)	41 (0.2)	89 (2.5)
2	4935 (22.2)	2905 (15.6)	2030 (56.1)
3	10 017 (45.1)	8553 (46.0)	1464 (40.5)
4	6177 (27.8)	6143 (33.0)	34 (0.9)
5	963 (4.3)	963 (5.2)	0
Race			
American Indian or Alaska Native	28 (0.1)	17 (0.1)	11 (0.3)
Asian	888 (4.0)	753 (4.0)	135 (3.7)
Black or African American	4383 (19.7)	3936 (21.2)	447 (12.4)
Native Hawaiian or Pacific Islander	35 (0.2)	29 (0.2)	6 (0.2)
White	8381 (37.7)	6361 (34.2)	2020 (55.8)
Other^c^	8507 (38.3)	7509 (40.4)	998 (27.6)
Ethnicity			
Hispanic or Latino	6799 (30.6)	6082 (32.7)	717 (19.8)
Not Hispanic or Latino	15 423 (69.4)	12 523 (67.3)	2900 (80.2)
Gender			
Women	10 639 (47.9)	8962 (48.2)	1677 (46.4)
Men	11 583 (52.1)	9643 (51.8)	1940 (53.6)

Abbreviations: BCH, Boston Children’s Hospital; ED, emergency department; MIMIC-IV, Medical Information Mart for Intensive Care IV.

^a^ There were 2472 patients (1.5%) who had no assigned ED triage acuity.

^b^ Patients can have more than 1 visit, each with different outcomes, so the unique number of admitted and discharged patients does not add up to the overall number of patients in the cohort.

^c^ Includes “unable to answer,” “declined to answer,” and “unknown.”

## Methods

We conducted a prognostic observational study of admission prediction models, trained on retrospective cohorts from 2 emergency medical centers. The study design was reviewed and approved with a waiver of informed consent by the Boston Children’s Hospital (BCH) institutional review board. Consent was waived because the protocol was determined to be exempt because it was limited to research activities in which the only involvement of human participants would be secondary research for which consent is not required. This study adheres to the Transparent Reporting of a Multivariable Prediction Model for Individual Prognosis or Diagnosis (TRIPOD) reporting guideline.

### Data Curation

We based our experiments on the task of inpatient hospital admission prediction for patients visiting the ED. Recently, multiple care centers have sought to develop, validate, and deploy ML models for this task, due to its significant effect on patient flow.^9,10,11,26^ The first ED site is sourced from the Medical Information Mart for Intensive Care IV (MIMIC-IV) ED database,^27^ a repository of deidentified ED visits to Beth Israel Deaconess Medical Center between January 1, 2011, and December 31, 2019, publicly released in 2021. The second is data collected from the BCH ED from June 1 to August 31, 2019. After data preprocessing (eMethods in Supplement 1), our analysis consisted of 160 016 visits by 90 005 unique patients in the MIMIC-IV cohort and 22 222 visits by 17 938 unique patients in the BCH cohort.

### Model Development

In both cohorts, we trained models to predict admission to an inpatient service among patients whose final disposition had yet to be decided. We used data collected during check-in (eg, chief concern), triage (eg, vital signs), patient clinical history (eg, number of previous admissions), and demographic data (self-reported race and ethnicity and gender). In the BCH cohort, we included additionally available data collected during the first 60 minutes of a patient’s stay, including laboratory test orders and medications (eTable 4 in Supplement 1).

We tested tree ensembles implemented in XGBoost and penalized logistic regression models (eMethods in Supplement 1). The hyperparameters of these models were tuned via halving grid search. In addition, we varied the fairness task and the patient subgroup definitions that were used in debiasing. These scenarios are summarized in the Box.

Box. **Experimental Setup for Assessing Algorithmic Fairness Under Intersectional and Marginal Fairness Scenarios**

**Variable**

Group construction scenarios

**Settings**

Base: no fairness optimization
Gender, race, ethnicity, ethnoracial: protect single attribute
Marginal: marginally protect all sensitive attributes
Intersectional: protect intersections of all sensitive attributes

**Variable**

Fairness task (algorithm)

**Settings**

Fair calibration (multicalibration boosting)
Fair false negative rate (fairness-oriented multiobjective optimization)

**Variable**

Base classifier

**Settings**

Penalized logistic regression (penalty: {ℓ1, ℓ2}, C: {0.01 . . . 10})
Random forest (n estimators: 100, max depth: 4)

**Variable**

Realizations

**Settings**

100 Trials of independent random seeds, 50/50 train/test split

### Fairness Approaches

For all models, we tested (1) multicalibration postprocessing to improve subgroup calibration performance and (2) FOMO to improve subgroup FNRs.

#### Multicalibration Postprocessing

Multicalibration postprocessing^24,28^ allows for the flexible specification of groups for marginal and intersectional fairness models. In brief, assume we have sample data (x*_i_, y_i_*), where x*_i_* is a vector of features and *y_i_* is a binary outcome for individual *i*. Let *C* represent a collection of subsets specified by protected attributes in x (ie, subgroups). For example, 1 subset in *C* could contain a subset of all patients who are Asian women. An α-multicalibrated model fulfills the constraint that among all subsets in *C* and binned prediction intervals (eg, predictions between [0.0, 0.1] or [0.7, 0.8]), the absolute difference between the expected outcome and expected prediction is at most α. The multicalibration algorithm updates model predictions until all groups defined by binned prediction intervals within collections in *C* with sample prevalence greater than γ satisfy the calibration error constraint α. Here, γ acts as a cutoff to prevent overfitting to very small groups. Our main results use α = .01 (constrain calibration error to 0.01) and γ = 0.001 (consider groups with 0.1% or higher sample prevalence). The eMethods in Supplement 1 contain a sensitivity analysis of these hyperparameters.

#### Fairness-Oriented Multiobjective Optimization

Achieving different notions of fairness in ML involves balancing the trade-off between error and fairness, where increased fairness may lead to higher error rates, and vice versa. Fairness-oriented multiobjective optimization optimizes this trade-off through multiobjective optimization, treating error and fairness as separate objectives.^25^

We used FOMO to jointly optimize the overall balanced accuracy of the models while minimizing the maximum FNRs among intersectional subgroups. This fairness definition has 2 motivations. First, it assumes that false discharges from the ED have the potential to cause more patient harm than a false admission. Second, unlike fairness metrics that optimize for FNR parity among groups (which can be achieved, for example, by making the model worse for some subgroups where it performs well), this metric focuses solely on improving the worst-case performance among patient subgroups. Minimizing subgroup FNRs must be balanced by minimizing overall FNRs and overall FPRs, which cause distributed harm to waiting patients due to overcrowding; hence, we jointly maximized for overall balanced accuracy. This design is intended to minimize potential harms that could result from simply equalizing FNRs and/or FPRs across subgroups, as discussed in other work.^29^

#### Protected Attributes and Intersectionality

The experiment in this study focuses on 3 protected attributes: race, ethnicity, and gender (in the MIMIC-IV cohort, race and ethnicity are reported as a combined ethnoracial variable). Scholars in the social, public health, and computational sciences^30,31^ have written about the importance of distinguishing between individual-level identities and systems of discrimination. Here, we conceptualized race, ethnicity, and gender attributes as imperfect proxies of systemic racism, systemic sexism, and structural barriers to health care access.^21^

### Statistical Analysis

Statistical analysis was conducted from January 2022 to August 2024. We conducted pairwise comparisons of expected calibration error (ECE) and FNR between pairs of modeling scenarios (eg, models trained with intersectional debiasing vs baseline models). The null hypothesis for each of these tests was that the observed scores could have been generated from the same modeling scenario. All reported *P* values on paired samples are the result of 2-sided Wilcoxon signed rank tests with Holm-Bonferroni correction. An adjusted *P* value of .05 was considered significant.

## Results

The analysis included 47 283 admissions from 160 016 visits (29.5%) from the MIMIC-IV cohort (mean [SD] age, 53.0 [19.3] years; 57.4% female patients and 42.6% male patients; 0.3% American Indian or Alaska Native patients, 3.7% Asian patients, 26.2% Black patients, 10.0% Hispanic or Latino patients, and 59.7% White patients) and 3617 admissions from 22 222 visits (16.3%) from BCH (mean [SD] age, 8.2 [6.8] years; 47.9% female patients and 52.1% male patients; 0.1% American Indian or Alaska Native patients, 4.0% Asian patients, 19.7% Black patients, 30.6% Hispanic or Latino patients, 0.2% Native Hawaiian or Pacific Islander patients, 37.7% White patients). Patient cohorts are detailed in the Table.

### Admission Rate Differences Among Patient Subgroups

We observed marked differences in admission rates by intersections of race, ethnicity, and gender (eTable 2 in Supplement 1), suggesting the importance of a performance-based fairness constraint (eg, calibration or error rates) as opposed to demographic parity, which equalizes outcome rates among subgroups and would therefore cause substantial deviations in predicted subgroup admission rates from what is observed.

### Fairness Without Accuracy Trade-Offs

**Figure 1.**
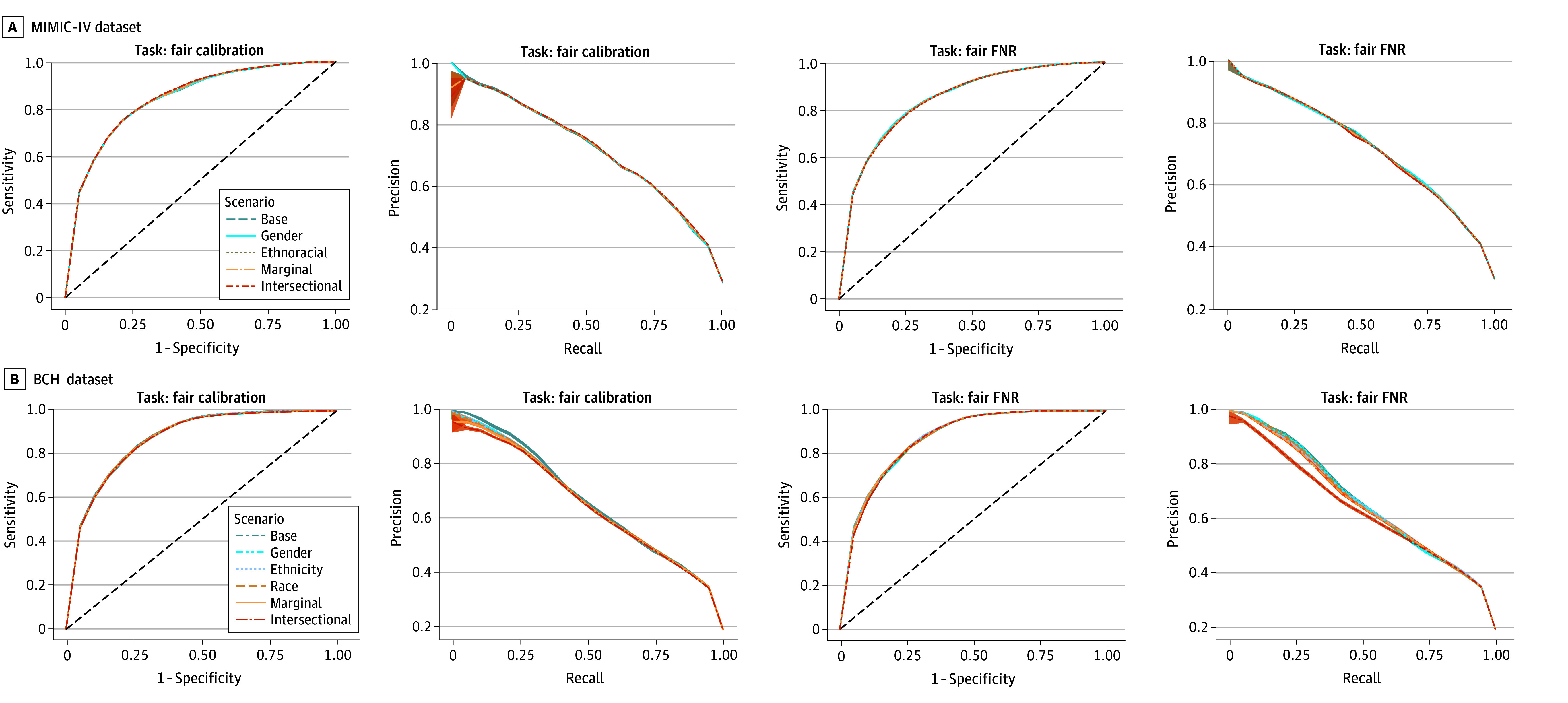
Performance of Debiased Models and Baseline Models Receiver operating characteristic curves and precision-recall curves for the prediction models on data from Medical Information Mart for Intensive Care IV (MIMIC-IV) (A) and Boston Children’s Hospital (BCH) (B). The left and right columns of subplots compare debiasing scenarios for fair calibration and fair false-negative rates (FNRs), respectively. In general, the fairness-aware models performed very similarly to the baseline models on the population as a whole.

The prevailing understanding of fairness as derived from the notions of equalized odds and parity among demographic groups is that they require trade-offs with overall accuracy,^32^ yet, in practice, such trade-offs may be negligible.^33^ Our findings were consistent with the latter; across both datasets and both fairness targets (calibration and FNR), debiasing methods had nearly identical overall performance to baseline models (mean area under the receiver operator characteristic curve [AUROC] within *±*0.01; Figure 1; eTable 3 in Supplement 1). Fairness improvements did not decrease overall accuracy compared with baseline models (eg, MIMIC-IV: mean [SD] AUROC, 0.85 [0.00], both models). When tasked with balancing FNRs in the BCH cohort, intersectionally debiased models exhibited a slightly lower area under the precision recall curve (AUPRC; base scenario mean [SD] AUPRC, 0.67 [0.01]; intersectional scenario mean [SD] AUPRC, 0.64 [0.02]). This difference is observed in BCH precision-recall curves in Figure 1, where the precision is lower for model thresholds with low recall or sensitivity, which occurs only when the model’s threshold for positive classification is set very high. For model thresholds corresponding to sensitivity or recall greater than 50%, intersectionally debiased models exhibit nearly identical precision to others (Figure 1).

### Fairness Gains With Intersectional Debiasing

**Figure 2.**
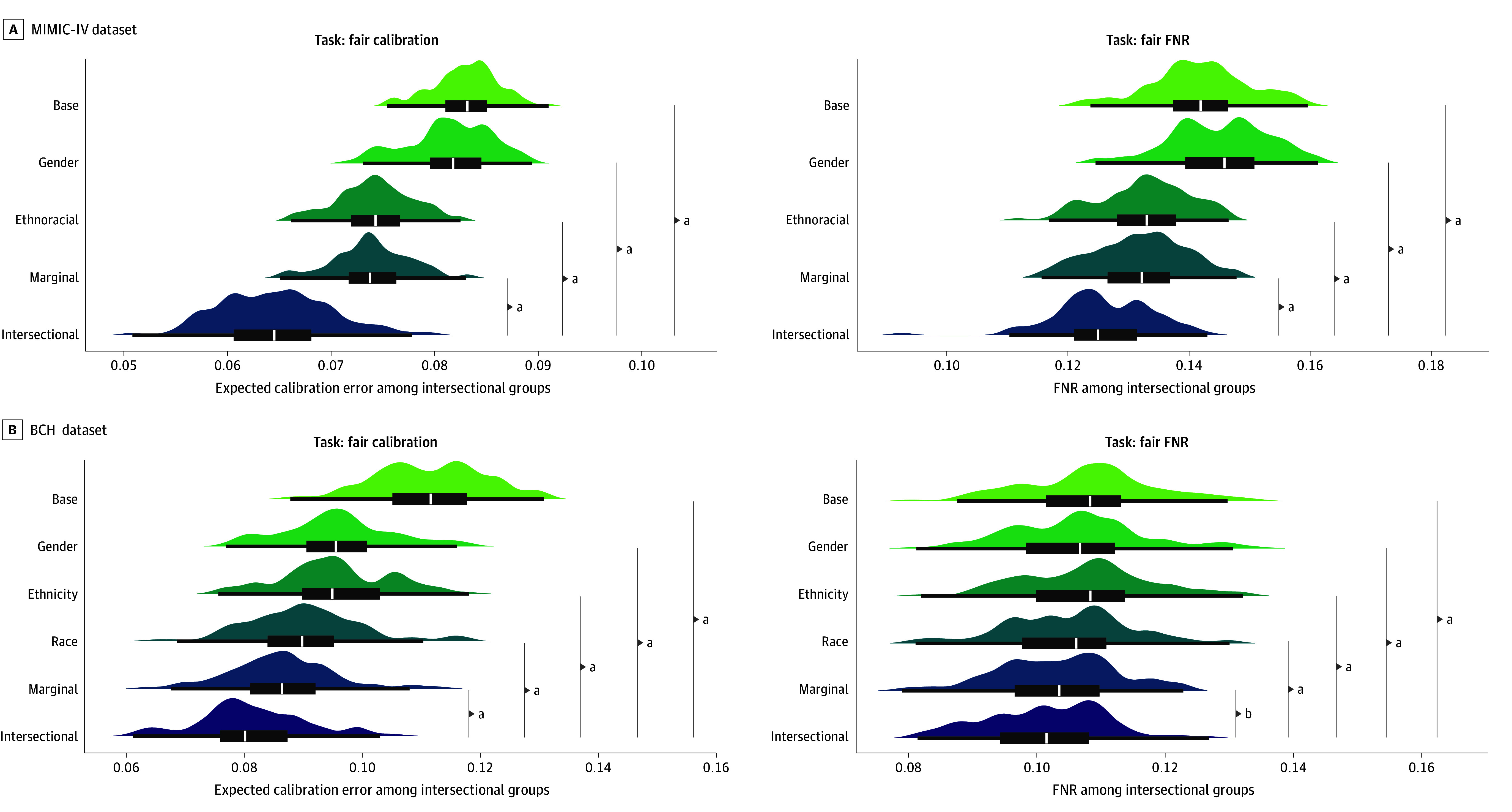
Intersectionally Debiased Models and Fairness for Intersectional Groups Beyond Marginally Debiased Models Fairness measures on test data across 100 trials under different debiasing scenarios for Medical Information Mart for Intensive Care IV (MIMIC-IV) (A) and Boston Children’s Hospital (BCH) (B). Left plots report the mean expected calibration error among intersectional groups when optimizing within-group calibration. Right plots report mean false-negative rate (FNR) among intersectional groups when optimizing groupwise FNRs. The scenarios (base, intersectional) are detailed in the Box. ^a^*P* < .001. ^b^b = 0.01 < *P* ≤ .05.

We compared the mean ECE and FNR among intersectional groups across debiasing conditions in Figure 2. Among MIMIC-IV groups, marginal fairness debiasing was associated with a reduced ECE from 0.083 to 0.074 (11.3%), while the fully intersectional approach was associated with a reduced ECE from 0.083 to 0.065 (22.3%; difference, 11.1%); among BCH groups, marginal fairness debiasing was associated with a reduced ECE from 0.111 to 0.086 (22.6%), whereas the fully intersectional approach was associated with a reduced ECE from 0.111 to 0.080 (28.3%; difference, 5.7%). Similarly, intersectional debiasing was associated with significantly lowered mean FNRs among intersectional groups compared with baseline (MIMIC-IV, from 0.142 to 0.125; 11.9% reduction; *P* < .001; BCH, from 0.108 to 0.102; 6.3% reduction; *P* < .001). Among MIMIC-IV groups, marginal debiasing was associated with lowered subgroup FNRs from 0.142 to 0.132 (6.8%). Debiasing based on ethnoracial group was associated with a larger singular reduction in error rates among intersectional groups than debiasing based on gender alone, but debiasing using the combination was associated with better performance than considering either attribute alone or additively.

### Intersectional Debiasing and Fairness for Small and Large Groups

#### Subgroup Calibration

In the MIMIC-IV cohort, compared with marginal debiasing, intersectional debiasing was associated with a significantly reduced ECE among American Indian or Alaska Native men (from 0.119 [95% CI, 0.060-0.163] to 0.097 [95% CI, 0.033-0.159]; *P* < .001), Asian men (from 0.091 [95% CI, 0.071-0.110] to 0.062 [95% CI, 0.034-0.090]; *P* < .001), Hispanic or Latino women (from 0.104 [95% CI, 0.089-0.110] to 0.088 [95% CI, 0.069-0.100]; *P* < .001), and Black or African American women (from 0.102 [95% CI, 0.095-0.107] to 0.089 [95% CI, 0.072-0.095]; *P* < .001). In the BCH cohort, compared with marginal debiasing, intersectional debiasing was associated with a significantly reduced ECE among White, Hispanic or Latino female patients (from 0.088 [95% CI, 0.057-0.128] to 0.076 [95% CI, 0.038-0.114]; *P* < .001).

#### Subgroup FNRs

In the MIMIC-IV cohort, compared with marginal debiasing, intersectional debiasing was associated with a significantly reduced FNR among American Indian or Alaska Native men (from 0.218 [95% CI, 0.165-0.289] to 0.206 [95% CI, 0.141-0.271]; *P* = .02), Asian men (from 0.205 [95% CI, 0.178-0.226] to 0.192 [95% CI, 0.168-0.221]; *P* < .001), Asian women (from 0.150 [95% CI, 0.134-0.167] to 0.144 [95% CI, 0.124-0.163]; *P* < .001), and White women (from 0.161 [95% CI, 0.145-0.174] to 0.153 [95% CI, 0.136-0.171]; *P* < .001). In the BCH cohort, compared with marginal debiasing, intersectional debiasing was associated with a significantly reduced FNR among American Indian or Alaska Native, non-Hispanic or non-Latino male patients (from 0.134 [95% CI, 0.000-0.317] to 0.103 [95% CI, 0.000-0.293]; *P* = .02).

**Figure 3.**
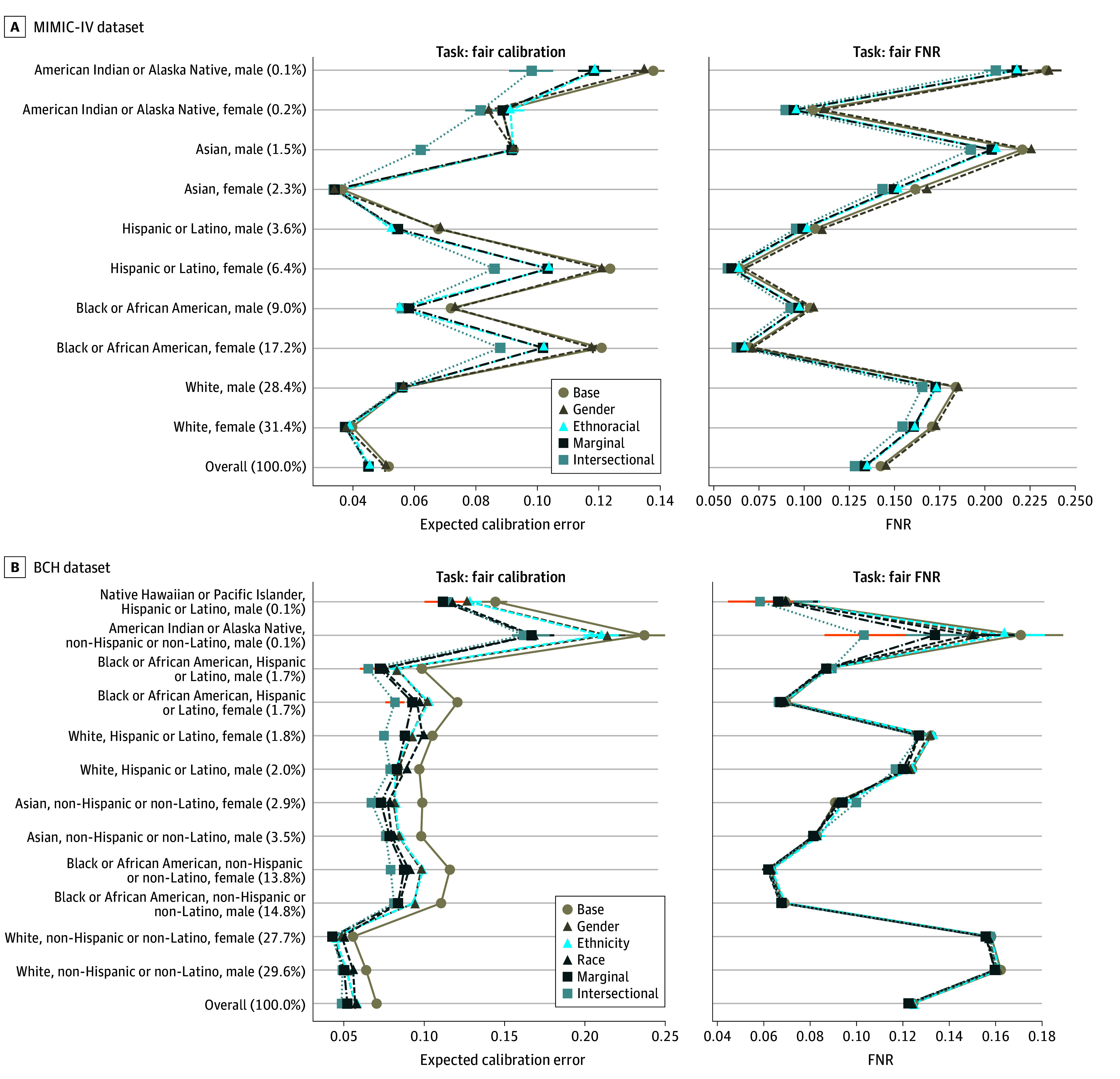
Model Performance on Each Intersectional Position According to Dataset and Fairness Consideration, Demarcated by Scenario A, Medical Information Mart for Intensive Care IV (MIMIC-IV) dataset. B, Boston Children’s Hospital (BCH) dataset. Points indicate bootstrap-estimated median performance over trials, and bars indicate the 95% CI on test data across 100 trials. FNR indicates false-negative rate.

In the MIMIC-IV cohort, intersectional debiasing approaches were associated with minimizing both the group-specific ECE (Figure 3) and the FNRs for the lowest prevalence group (American Indian or Alaska Native men; prevalence = 0.1%) and highest prevalence group (White women; prevalence = 31.4%) more than other methods (*P* <.001). We further investigated the association of group size with multicalibration postprocessing in eFigure 2 in Supplement 1. Results were largely consistent for logistic regression models (eFigures 3 and 4 in Supplement 1). Trade-offs between FNR and accuracy for FOMO-trained models are shown in eFigure 5 in Supplement 1.

## Discussion

To date, most model bias is identified after deployment,^34^ with few clinical prediction models incorporating fairness notions during development. To our knowledge, this study is among the first to implement an intersectional debiasing approach for clinical prediction models and demonstrate that (1) it can significantly improve the performance of a model on subgroups vs the more common marginal approaches and (2) it can reduce unfairness with minor changes in overall performance. In the MIMIC-IV cohort, intersectionally debiased ML models were associated with a 22.3% reduction in subgroup ECE and a 11.3% reduction in subgroup FNR with no change in AUROC or AUPRC. In the BCH cohort, models were associated with a 28.3% reduction in subgroup ECE with no reduction in AUROC or AUPRC and a 6.3% reduction in subgroup FNR for no reduction in AUROC and a 4.5% reduction in mean AUPRC (concentrated at low sensitivity model thresholds).

### Limitations

This study has some limitations. A challenge of intersectional approaches using demographic traits is that as more protected attributes are added, group sizes shrink. We limited our analysis to 3 attributes: race, ethnicity, and sex, and only considered intersectional groups representing at least 0.1% of the population. Although multicalibration handles small group sizes with a threshold, other fairness methods use a prior probability for group outcomes. We tested both approaches in FOMO and found no significant association with results. Future studies could explore additional attributes and larger datasets to examine the limits of fairness gains for smaller intersectional groups.

Our results are limited to 1 clinically relevant prediction problem, but it is a type of resource allocation problem that is widely found in clinical settings. Further work should examine the extent to which our observations are generalizable to other settings of interest.

We did not answer the question of whether subgroup calibration or subgroup FNR is a more important fairness consideration for this task; instead, we measured the importance of intersectional debiasing for multiple scenarios. Calibration is important for interpreting risk scores and doing risk stratification. False-negative rates are important for interpreting the risk of missed interventions. False-negative rates, FPRs, and calibration cannot be simultaneously equal when subgroups exhibit different prevalences of the outcome.^23^ Future studies could consider the trade-offs of these fairness metrics that are not covered here. Similarly, future prospective studies depend on engagement with community stakeholders to define which metrics are more important in clinical decision support.

## Conclusions

In this prognostic study of ED admission prediction models, we found that intersectional subgroups were important to include during the model development process to achieve the best subgroup calibration in validation data. These fairness gains were obtained without sacrificing overall model performance, which suggests that clinical risk prediction models should consider incorporating intersectional debiasing into their development.

## Notes

Supplement 1. **eFigure 1.** An Illustration of the Admission Prediction Task and Its Utility in the Emergency Department (ED) During the Typical Timeline of a Patient Visit **eTable 1.** Properties of a Number of Algorithms Proposed for Fair Machine Learning, Along With Their Properties and Support for Intersectional Fairness Definitions **eTable 2.** Fraction of Emergency Admissions (%) by Intersectional Position for Patients in the MIMIC-IV (Top) and BCH (Bottom) Cohorts **eTable 3.** The Mean (± Standard Deviation Over 100 Trials) Area Under the ROC Curve (AUROC) and Precision-Recall Curve (AUPRC) of Prediction Models by Dataset, Fairness Task, and Modeling Scenario, Corresponding to the Curves in Figure 1
eMethods.
**eTable 4.** Features Used for Emergency Admission Prediction in the MIMIC-IV and BCH Cohorts **eFigure 2.** Intersectional Group-Wise Expected Calibration Error on MIMIC-IV as a Function of *γ* (Row), *α* (Column), Base ML Model (X-Axis), and Optimization Scenario (Color) **eFigure 3.** Expected Calibration Error (ECE) as a Function of Group Prevalence for LR Models Trained on MIMIC-IV, Under Different Combinations of *α* and *γ* **eFigure 4.** False Negative Rates (FNR) Among Intersectional Groups Under Different Base Models (Left: Random Forests (RF), Right: Penalized Logistic Regression (LR)) and FOMO De-Biasing Scenarios (Y-Axis) for MIMIC-IV (Top) and BCH (Bottom) **eFigure 5.** Accuracy-Fairness Trade-Offs and Model Selection
eReferences.

Supplement 2.
Data Sharing Statement

## References

[1] Parast L, Mathews M, Martino S, Lehrman WG, Stark D, Elliott MN. Racial/ethnic differences in emergency department utilization and experience. J Gen Intern Med. 2022;37(1):49-56. doi:10.1007/s11606-021-06738-0 PMC8021298 33821410

[2] Islami F, Guerra CE, Minihan A, et al. American Cancer Society’s report on the status of cancer disparities in the United States, 2021. CA: Cancer J Clin. 2022;72(2):112-143. doi:10.3322/caac.21703 34878180

[3] He J, Zhu Z, Bundy JD, Dorans KS, Chen J, Hamm LL. Trends in cardiovascular risk factors in US adults by race and ethnicity and socioeconomic status, 1999-2018. JAMA. 2021;326(13):1286-1298. doi:10.1001/jama.2021.15187 PMC8493438 34609450

[4] Mohamud MA, Campbell DJT, Wick J, et al. 20-Year trends in multimorbidity by race/ethnicity among hospitalized patient populations in the United States. Int J Equity Health. 2023;22(1):137. doi:10.1186/s12939-023-01950-2 PMC10367428 37488549

[5] Lu FQ, Hanchate AD, Paasche-Orlow MK. Racial/ethnic disparities in emergency department wait times in the United States, 2013-2017. Am J Emerg Med. 2021;47:138-144. doi:10.1016/j.ajem.2021.03.051 33812329

[6] Pines JM, Russell LA, Hollander JE. Racial disparities in emergency department length of stay for admitted patients in the United States. Acad Emerg Med. 2009;16(5):403-410. doi:10.1111/j.1553-2712.2009.00381.x 19245372

[7] Chalfin DB, Trzeciak S, Likourezos A, Baumann BM, Dellinger RP; DELAY-ED study group. Impact of delayed transfer of critically ill patients from the emergency department to the intensive care unit. Crit Care Med. 2007;35(6):1477-1483. doi:10.1097/01.CCM.0000266585.74905.5A 17440421

[8] Carr BG, Kaye AJ, Wiebe DJ, Gracias VH, Schwab CW, Reilly PM. Emergency department length of stay: a major risk factor for pneumonia in intubated blunt trauma patients. J Trauma. 2007;63(1):9-12. doi:10.1097/TA.0b013e31805d8f6b 17622862

[9] Barak-Corren Y, Israelit SH, Reis BY. Progressive prediction of hospitalisation in the emergency department: uncovering hidden patterns to improve patient flow. Emerg Med J. 2017;34(5):308-314. doi:10.1136/emermed-2014-203819 28188202

[10] Yuval BC, Fine AM, Reis BY. Early prediction model of patient hospitalization from the pediatric emergency department. Pediatrics. 2017;139(5):e20162785. doi:10.1542/peds.2016-2785 28557729

[11] Barak-Corren Y, Agarwal I, Michelson KA, . Prediction of patient disposition: comparison of computer and human approaches and a proposed synthesis. J Am Med Inform Assoc. 2021;28(8):1736-1745. doi:10.1093/jamia/ocab076 34010406 PMC8324238

[12] Lett E, La Cava WG. Translating intersectionality to fair machine learning in health sciences. Nat Mach Intell. 2023;5(5):476-479. doi:10.1038/s42256-023-00651-3 37600144 PMC10437125

[13] Kearns M, Neel S, Roth A, Wu ZS. Preventing fairness gerrymandering: auditing and learning for subgroup fairness. *arXiv*. Preprint posted online December 3, 2018. doi:10.48550/arXiv.1711.05144

[14] Kearns M, Neel S, Roth A, Wu ZS. An empirical study of rich subgroup fairness for machine learning. *arXiv*. Preprint posted online August 24, 2018. doi:10.48550/arXiv.1808.08166

[15] Crenshaw K. Demarginalizing the intersection of race and sex: a black feminist critique of antidiscrimination doctrine, feminist theory and antiracist politics. *University of Chicago Legal Forum*. Vol 1989. Issue 1, Article 8. The University of Chicago Law School. Chicago Unbound. Accessed April 21, 2025. https://chicagounbound.uchicago.edu/uclf/vol1989/iss1/8/

[16] Crenshaw K. Mapping the margins: intersectionality, identity politics, and violence against women of color. Stanford Law Rev. 1991;43(6):1241-1299. doi:10.2307/1229039

[17] Collins PH. Black feminist thought in the matrix of domination. In: *Black Feminist Thought: Knowledge, Consciousness, and the Politics of Empowerment*. UnWin Hyman; 1990:221-238.

[18] Ange-Marie H. Intersectionality: An Intellectual History. Oxford University Press; 2016.

[19] Combahee River Collective. *The Combahee River Collective Statement: Black Feminist Organizing in the Seventies and Eighties*. Kitchen Table: Women of Color Press; 1986.

[20] Truth S. “Ain’t I a Woman?” (1851). In: Owens L III, Bishop T, Ortolano S. *Starting the Journey: An Intro to College Writing*. Accessed April 21, 2025. https://fsw.pressbooks.pub/enc1101/chapter/sojourner-truth-aint-i-a-woman-1851/

[21] Lett E, Asabor E, Beltrán S, Cannon AM, Arah OA. Conceptualizing, contextualizing, and operationalizing race in quantitative health sciences research. Ann Fam Med. 2022;20(2):157-163. doi:10.1370/afm.2792 35045967 PMC8959750

[22] Barocas S, Hardt M, Narayanan A. *Fairness and Machine Learning: Limitations and Opportunities*. MIT Press; 2023. Accessed April 21, 2025. https://fairmlbook.org/

[23] Kleinberg J, Mullainathan S, Raghavan M. Inherent trade-offs in the fair determination of risk scores. *arXiv*. Published online September 19, 2016. doi:10.48550/arXiv.1609.05807

[24] Pfisterer F, Kern C, Dandl S, Sun M, Kim MP, Bischl B. mcboost: Multi-calibration boosting for R. J Open Source Softw. 2021;6(64):3453. doi:10.21105/joss.03453

[25] La Cava William G. Optimizing fairness tradeoffs in machine learning with multiobjective meta-models. In: *GECCO ’23: Proceedings of the Genetic and Evolutionary Computation Conference*. Association for Computing Machinery; 2023:511-519. doi:10.1145/3583131.3590487

[26] Barak-Corren Y, Chaudhari P, Perniciaro J, Waltzman M, Fine AM, Reis BY. Prediction across healthcare settings: a case study in predicting emergency department disposition. NPJ Digit Med. 2021;4(1):169. doi:10.1038/s41746-021-00537-x 34912043 PMC8674364

[27] Johnson A, Bulgarelli L, Pollard T, et al Celi LA, Mark R, Horng S. MIMIC-IV-ED (version 2.2). PhysioNet. 2023. Accessed April 21, 2025. https://physionet.org/content/mimic-iv-ed/2.2/

[28] Hebert-Johnson U, Kim M, Reingold O, Rothblum G. Multicalibration: calibration for the (computationally-identifiable) masses. In: *Proceedings of the 35th International Conference on Machine Learning*. Vol 80. PMLR; 2018:1939-1948.

[29] Chohlas-Wood A, Coots M, Goel S, Nyarko J. Designing equitable algorithms. Nat Comput Sci. 2023;3(7):601-610. doi:10.1038/s43588-023-00485-4 38177749

[30] Bowleg L. The problem with the phrase women and minorities: intersectionality—an important theoretical framework for public health. Am J Public Health. 2012;102(7):1267-1273. doi:10.2105/AJPH.2012.300750 PMC3477987 22594719

[31] Hanna A, Denton E, Smart A, Smith-Loud J. Towards a critical race methodology in algorithmic fairness. In: *FAT* ’20: Proceedings of the 2020 Conference on Fairness, Accountability, and Transparency*. Association for Computing Machinery; 2020:501-512. doi:10.1145/3351095.3372826

[32] Pleiss G, Raghavan M, Wu F, Kleinberg J, Weinberger KQ. On fairness and calibration. In: Guyon I, Von Luxburg U, Bengio S, et al, eds. *Advances in Neural Information Processing Systems 30*. Curran Associates Inc; 2017:5680-5689.

[33] Rodolfa KT, Hemank L, Rayid G. Empirical observation of negligible fairness—accuracy trade-offs in machine learning for public policy. Nat Mach Intell. 2021;3:896-904. doi:10.1038/s42256-021-00396-x

[34] Obermeyer Z, Powers B, Vogeli C, Mullainathan S. Dissecting racial bias in an algorithm used to manage the health of populations. Science. 2019;366(6464):447-453. doi:10.1126/science.aax2342 31649194

