# Supplementary material for: Intersectional and Marginal Debiasing in Prediction Models for Emergency Admissions
Source: JAMA Netw Open. 2025 May 29;8(5):e2512947. doi: 10.1001/jamanetworkopen.2025.12947 (PMC12123471; doi:10.1001/jamanetworkopen.2025.12947)
Supplement: Supplement 1. — eFigure 1. An Illustration of the Admission Prediction Task and Its Utility in the Emergency Department (ED) During the Typical Timeline of a Patient Visit eTable 1. Properties of a Number of Algorithms Proposed for Fair Machine Learning, Along With Their Properties and Support for Intersectional Fairness Definitions eTable 2. Fraction of Emergency Admissions (%) by Intersectional Position for Patients in the MIMIC-IV (Top) and BCH (Bottom) Cohorts eTable 3. The Mean (± Standard Deviation Over 100 Trials) Area Under the ROC Curve (AUROC) and Precision-Recall Curve (AUPRC) of Prediction Models by Dataset, Fairness Task, and Modeling Scenario, Corresponding to the Curves in Figure 1 eMethods. eTable 4. Features Used for Emergency Admission Prediction in the MIMIC-IV and BCH Cohorts eFigure 2. Intersectional Group-Wise Expected Calibration Error on MIMIC-IV as a Function of γ (Row), α (Column), Base ML Model (X-Axis), and Optimization Scenario (Color) eFigure 3. Expected Calibration Error (ECE) as a Function of Group Prevalence for LR Models Trained on MIMIC-IV, Under Different Combinations of α and γ eFigure 4. False Negative Rates (FNR) Among Intersectional Groups Under Different Base Models (Left: Random Forests (RF), Right: Penalized Logistic Regression (LR)) and FOMO De-Biasing Scenarios (Y-Axis) for MIMIC-IV (Top) and BCH (Bottom) eFigure 5. Accuracy-Fairness Trade-Offs and Model Selection eReferences. [file jamanetwopen-e2512947-s001.pdf]

Supplementary Online Content

Lett E, Shahbandegan S, Barak-Corren Y, Fine AM, La Cava WG. Intersectional and marginal debiasing in prediction models for emergency admissions. *JAMA Netw Open.* 2025;8(5):e2512947. doi:10.1001/jamanetworkopen.2025.12947

eFigure 1. An Illustration of the Admission Prediction Task and Its Utility in the Emergency Department (ED) During the Typical Timeline of a Patient Visit
eTable 1. Properties of a Number of Algorithms Proposed for Fair Machine Learning, Along With Their Properties and Support for Intersectional Fairness Definitions
eTable 2. Fraction of Emergency Admissions (%) by Intersectional Position for Patients in the MIMIC-IV (Top) and BCH (Bottom) Cohorts
eTable 3. The Mean (± Standard Deviation Over 100 Trials) Area Under the ROC Curve (AUROC) and Precision-Recall Curve (AUPRC) of Prediction Models by Dataset, Fairness Task, and Modeling Scenario, Corresponding to the Curves in Figure 1
eMethods.
eTable 4. Features Used for Emergency Admission Prediction in the MIMIC-IV and BCH Cohorts
eFigure 2. Intersectional Group-Wise Expected Calibration Error on MIMIC-IV as a Function of γ (Row), α (Column), Base ML Model (X-Axis), and Optimization Scenario (Color)
eFigure 3. Expected Calibration Error (ECE) as a Function of Group Prevalence for LR Models Trained on MIMIC-IV, Under Different Combinations of α and γ
eFigure 4. False Negative Rates (FNR) Among Intersectional Groups Under Different Base Models (Left: Random Forests (RF), Right: Penalized Logistic Regression (LR)) and FOMO De-Biasing Scenarios (Y-Axis) for MIMIC-IV (Top) and BCH (Bottom)
eFigure 5. Accuracy-Fairness Trade-Offs and Model Selection
eReferences.

This supplementary material has been provided by the authors to give readers additional information about their work.

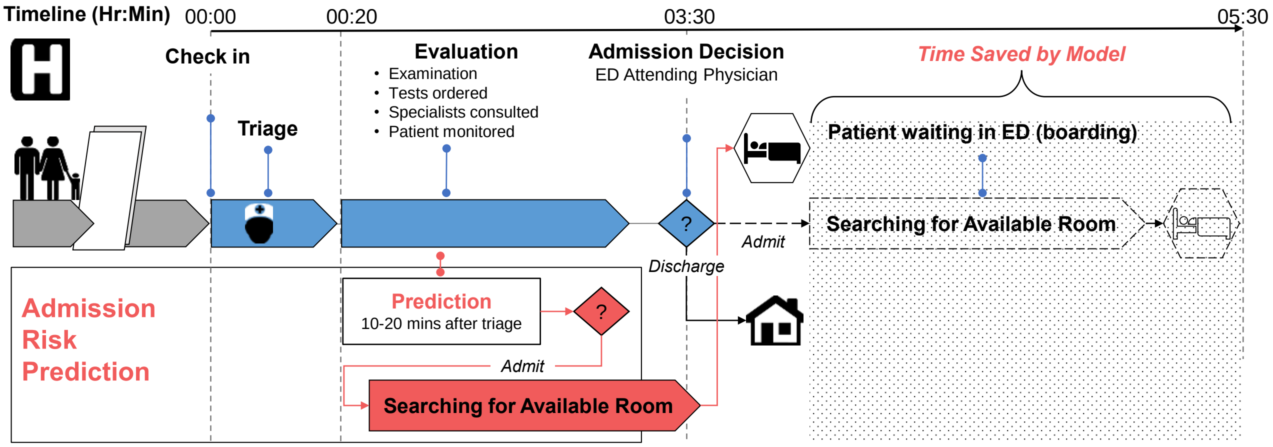

eFigure 1. An Illustration of the Admission Prediction Task and Its Utility in the Emergency Department (ED) During the Typical Timeline of a Patient Visit. Normally, patients who will be admitted wait while care coordinators find an available room (known as boarding). Admission prediction algorithms flag high risk patients early in the visit so that the bed coordination can happen before the ED attending physician makes an admission decision for the patient.

© 2025 Lett E et al. *JAMA Network Open.*

eTable 1. Properties of a Number of Algorithms Proposed for Fair Machine Learning, Along With Their Properties and Support for Intersectional Fairness Definitions. DP: Demographic Parity; FNR: False Negative Rate; FPR: false positive rate. Model-Agnostic indicates that the algorithm supports many common base ML models. The algorithms in bold are the two used in this study.

Stage	Algorithm	Fairness Definition			Intersectional Groups	Model-Agnostic
		DP	FNR/FPR	Calibration		
Pre	Reweighing [1]	✓				✓
	Fair Feature Selection [2]	✓				✓
Train	Adversarial Debiasing [3]		✓			
	Differential Fairness [4]	✓			✓	
	Exponentiated Gradients [5]	✓	✓			✓
	GerryFair [6]	✓	✓		✓	✓
	**FOMO** [7]	✓	✓	✓	✓	✓
Post	Threshold Optimization [8]	✓	✓		✓	
	Calibrated Equalized Odds [9]		✓	✓		✓
	**MultiCalibration** [10]			✓	✓	✓
	MultiAccuracy [11]		✓		✓	✓

© 2025 Lett E et al. *JAMA Network Open.*

eTable 2. Fraction of Emergency Admissions (%) by Intersectional Position for Patients in the MIMIC-IV (Top) and BCH (Bottom) Cohorts. AI/AN: American Indian / Alaskan Native; AA: African American; NHPI: Native Hawaiian Pacific Islander; (N)HL: (Not) Hispanic/Latino; F: female; M: male. Subgroups with fewer than five samples are omitted.

**MIMIC-IV ED**			
**Gender**	**Female**	**Male**	**Overall**
**Ethnoracial Group**			
AI/AN	70 / 257 (27)	82 / 170 (48)	152 / 427 (36)
ASIAN	1,043 / 3,595 (29)	1,032 / 2,384 (43)	2,075 / 5,979 (35)
BLACK/AA	3,124 / 27,486 (11)	2,603 / 14,458 (18)	5,727 / 41,944 (14)
HL	1,063 / 10,262 (10)	1,168 / 5,795 (20)	2,231 / 16,057 (14)
WHITE	18,147 / 50,174 (36)	18,951 / 45,435 (42)	37,098 / 95,609 (39)
Overall	23,447 / 91,774 (26)	23,836 / 68,242 (35)	47,283 / 160,016 (30)

**BCH ED**				
	**Gender**	**Female**	**Male**	**Overall**
**Race**	**Ethnicity**			
AI/AN	HL	-	-	-
	NHL	1 / 5 (20)	10 / 19 (53)	11 / 24 (46)
	Overall	1 / 7 (14)	10 / 21 (48)	11 / 28 (39)
ASIAN	HL	-	-	-
	NHL	61 / 398 (15)	74 / 484 (15)	135 / 882 (15)
	Overall	61 / 401 (15)	74 / 487 (15)	135 / 888 (15)
BLACK/AA	HL	22 / 232 (9)	25 / 231 (11)	47 / 463 (10)
	NHL	188 / 1,892 (10)	212 / 2,028 (10)	400 / 3,920 (10)
	Overall	210 / 2,124 (10)	237 / 2,259 (10)	447 / 4,383 (10)
NHPI	HL	2 / 9 (22)	1 / 15 (7)	3 / 24 (12)
	NHL	2 / 7 (29)	1 / 4 (25)	3 / 11 (27)
	Overall	4 / 16 (25)	2 / 19 (11)	6 / 35 (17)
Other	HL	261 / 2,812 (9)	308 / 2,963 (10)	569 / 5,775 (10)
	NHL	182 / 1,232 (15)	247 / 1,500 (16)	429 / 2,732 (16)
	Overall	443 / 4,044 (11)	555 / 4,463 (12)	998 / 8,507 (12)
WHITE	HL	53 / 247 (21)	45 / 280 (16)	98 / 527 (19)
	NHL	905 / 3,800 (24)	1,017 / 4,054 (25)	1,922 / 7,854 (24)
	Overall	958 / 4,047 (24)	1,062 / 4,334 (25)	2,020 / 8,381 (24)
Overall	HL	338 / 3,305 (10)	379 / 3,494 (11)	717 / 6,799 (11)
	NHL	1,339 / 7,334 (18)	1,561 / 8,089 (19)	2,900 / 15,423 (19)
	Overall	1,677 / 10,639 (16)	1,940 / 11,583 (17)	3,617 / 22,222 (16)

© 2025 Lett E et al. *JAMA Network Open.*

eTable 3. The Mean (± Standard Deviation Over 100 Trials) Area Under the ROC Curve (AUROC) and Precision-Recall Curve (AUPRC) of Prediction Models by Dataset, Fairness Task, and Modeling Scenario, Corresponding to the Curves in Figure 1. In general, the fairness-aware models perform very similarly to the baseline models.

MIMIC-IV		Scenario				
**Metric**	**Task**	**Base**	**Gender**	**Ethnoracial**	**Marginal**	**Intersectional**
**AUPRC**	**Fair Calibration**	0.72±0.00	0.72±0.00	0.72±0.00	0.72±0.00	0.72±0.00
	**Fair FNR**	0.72±0.00	0.72±0.00	0.72±0.00	0.72±0.00	0.72±0.00
**AUROC**	**Fair Calibration**	0.85±0.00	0.85±0.00	0.85±0.00	0.85±0.00	0.85±0.00
	**Fair FNR**	0.85±0.00	0.85±0.00	0.85±0.00	0.85±0.00	0.84±0.00

BCH		Scenario					
**Metric**	**Task**	**Base**	**Gender**	**Ethnicity**	**Race**	**Marginal**	**Intersectional**
**AUPRC**	**Fair Calibration**	0.67±0.01	0.66±0.01	0.66±0.01	0.66±0.01	0.66±0.01	0.65±0.01
	**Fair FNR**	0.67±0.01	0.67±0.01	0.66±0.01	0.66±0.01	0.66±0.01	0.64±0.02
**AUROC**	**Fair Calibration**	0.88±0.01	0.88±0.01	0.88±0.01	0.88±0.01	0.88±0.01	0.87±0.01
	**Fair FNR**	0.88±0.01	0.88±0.01	0.88±0.01	0.88±0.01	0.88±0.01	0.87±0.01

© 2025 Lett E et al. *JAMA Network Open.*

eMethods.

Additional Experiment Details

Data preprocessing and cleaning. For numeric data in the MIMIC-IV-ED triage table (Table S4), we encoded outliers as `NaNs` according to the following (min,max) ranges: temperature (95-105 F); heart rate (30-300 beats per minute); respiratory rate (2-200 breaths per minute), oxygen saturation (50-100%); systolic blood pressure (30-400 mmHg), diastolic blood pressure (30-300 mmHg); pain scores (0-20); acuity score (1-5).

For both cohorts, chief complaint consists of brief strings of free text. For these data, we first applied simple harmonization and cleaning heuristics and then one-hot- encoded the result, filtering out tokens ocurring less than 1% of the time. In our preliminary analysis we evaluated the use of pre-trained word embeddings for chief complaint but did not find that they improved performance versus one-hot-encoding.

The BCH database contained the following race options: 'Black or African American'; 'White'; 'Other'; 'Unable to Answer'; 'Declined to Answer'; 'Unknown'; 'Asian' 'American Indian or Alaska Native'; 'Native Hawaiian or Other Pacific Islander'; 'Hispanic or Latino'. The "Other" category was pre-processed to contain 'Unable to Answer', 'Declined to Answer', and 'Unknown'. The BCH database also contained the following ethnicity options, recorded in response to "Hispanic Yes or No": 'No'; 'Yes'; 'Unknown'; 'Declined to Answer'; 'UNAVAILABLE'; 'DECLINED'; 'SOURCE NOT DEFINED'. Patients who wrote Hispanic or Latino for race had these entries mapped to their ethnicity values. MIMIC-IV ethnoracial data matches what was shown here, with the additional option of 'Unknown/Unable to Obtain', which was rolled into 'Other' for our analyses.

Features used in training prediction models. Table S4 details the features used for emergency admission prediction that are derived from the available electronic health records in the two patient cohorts.

Algorithm Implementation. We use a Python implementation of Multicalibration Boosting available from github.com/cavalab/pmcboost and derived from La Cava et al [14]. Fairness-Oriented Multiobjective Optimization (FOMO) is available from cavalab.org/fomo. FOMO serves as a generic interface between the multi-objective optimization algorithms from pymoo and ML methods that follow the scikit-learn API while accepting sample weights as an argument during training (i.e. in calls to `fit()`). Our experimental study focuses on utilizing the popular NSGA2 [15] algorithm in conjunction with two widely used ML methods that support weighted classification: random forests (implemented in XGBoost) and penalized linear regression (implemented in scikit-learn [16]). The code to run the experiments is available from the repository github.com/cavalab/marginal-intersectional.

Training. We ran 100 trials of each combination of dataset (MIMIC-IV, BCH), fairness task (fair calibration, fair false negative rates), group construction scenario (Base, Race, Gender, Ethnicity, Marginal, Intersectional), and base model (penalized logistic regression, random forests), as shown in Table 2. Each trial utilized a unique random seed that resulted in a random shuffle of the data which was split into 50% train/ 50% test sets. Splits were stratified by

© 2025 Lett E et al. *JAMA Network Open.*

outcome (admission), gender, and race to maintain appropriate representation in each. For the runs using FOMO and MIMIC-IV data, the training set was further reduced to 10% (approximately 16k patients) to reduce computation time.

Additional Experiments

In this section we report additional experiments meant to characterize the sensitivity of the studied fairness algorithms to hyperparameters and design variables. For both multicalibration boosting and FOMO, we analyze how the choice of base ML model, group prevalence, and dataset affect the results. In the case of multicalibration boosting, we studied the choice of α, a termination criteria that defines the group-specific calibration error threshold, and γ, a parameter that controls the minimum prevalence of a group to be considered for updating. In the case of FOMO, we looked at the effect of using a weighted subgroup FNR metric that accounts for prior probability of the groups, and the effect of a fairness meta-model complexity.

© 2025 Lett E et al. *JAMA Network Open.*

eTable 4. Features Used for Emergency Admission Prediction in the MIMIC-IV and BCH Cohorts. The BCH data includes a larger set of predictors (n = 155, BCH; n = 60, MIMIC-IV) including indicators of laboratory tests and a larger set of reported symptoms beyond chief complaint. HR: heart rate; RR: respiratory rate; SBP: systolic blood pressure; DBP: diastolic blood pressure; BMI: body mass index.

Description	Features
	MIMIC-IV
Vitals	temperature, HR, RR, oxygen saturation, SBP, DBP
Triage Acuity	Emergency Severity Index[12]
Check-in Data	chief complaint, self-reported pain score
Health Record Data	no. previous visits, no. previous admissions
Demographic Data	ethnoracial group, gender, age
	BCH
Demographic Data	Gender, Race, Ethnicity, Age
Check-in Data	ED Day Of Week, ED Checkin Month, ED Checkin Year, ED Arrival Mode, Weekend, Miles Traveled, Patient State of Residence
Triage Data	Emergency Severity Index, ED Teams, ED Room Type, chief complaint, pain count, pain max, pain increase, CHEWS [13] count, CHEWS max, CHEWS increase
Medications	acetaminophen, albuterol, dexamethasone, epinephrine/lidocaine/tetracaine topical, ibuprofen, ipratropium, ondansetron, Sodium Chloride 0.9%, gastro count, gastro max, gastro increase, time till first med, drugs count, medication route (inhaled, intravenous, nebulized, oral, topical)
Vitals	HR count, HR min, HR max, HR sd, HR mean, RR count, RR min, RR max, RR sd, RR mean, temp low, temp normal, temp high low, temp not taken
Lab Test Orders	labs count, time till first lab, Blood Culture Routine, Aerobic, Blood Culture, Aerobic and Anaerobic, Blood Gas, Venous, C-Reactive Protein, Calcium, Plasma, Chemistry Panel, Chemistry Extended Panel, Complete Blood Count with Differential, Differential, Automated, Drug Screen, Urine (drugs of abuse), Erythrocyte Sedimentation Rate, Lipase, Plasma, Liver Function Tests, Urinalysis, Dipstick, Urine Culture, time till first test, tests count, Chest X-ray
Symptoms	Abdominal pain, Attention deficit disorder with hyperactivity, Allergic rhinitis, Anx- iety, Asthma, Atopic eczema, Autistic disorder, Chronic constipation, Constipation, Cough, Dental caries, Depression, Dermatitis, Developmental delay, Dysphagia, Epilepsy, Eustachian tube dysfunction, Feeding problem, Fever, Food allergy, Gas- troesophageal reflux, Global developmental delay, Headaches, Hypotonia, Obesity, Obstructive sleep apnea, Oropharyngeal dysphagia, Other Problem, Patent ductus arteriosus, Patent foramen ovale, Pediatric BMI greater than or equal to 95th percentile, Prematurity, Recurrent acute otitis media, Seizure, Snoring, Speech delay, Tonsillar and adenoid hypertrophy, Tonsils hypertrophy, Vitamin D deficiency, Vomiting
Clinical History	problems count, prior visits, prior admissions, admission ratio

© 2025 Lett E et al. *JAMA Network Open.*

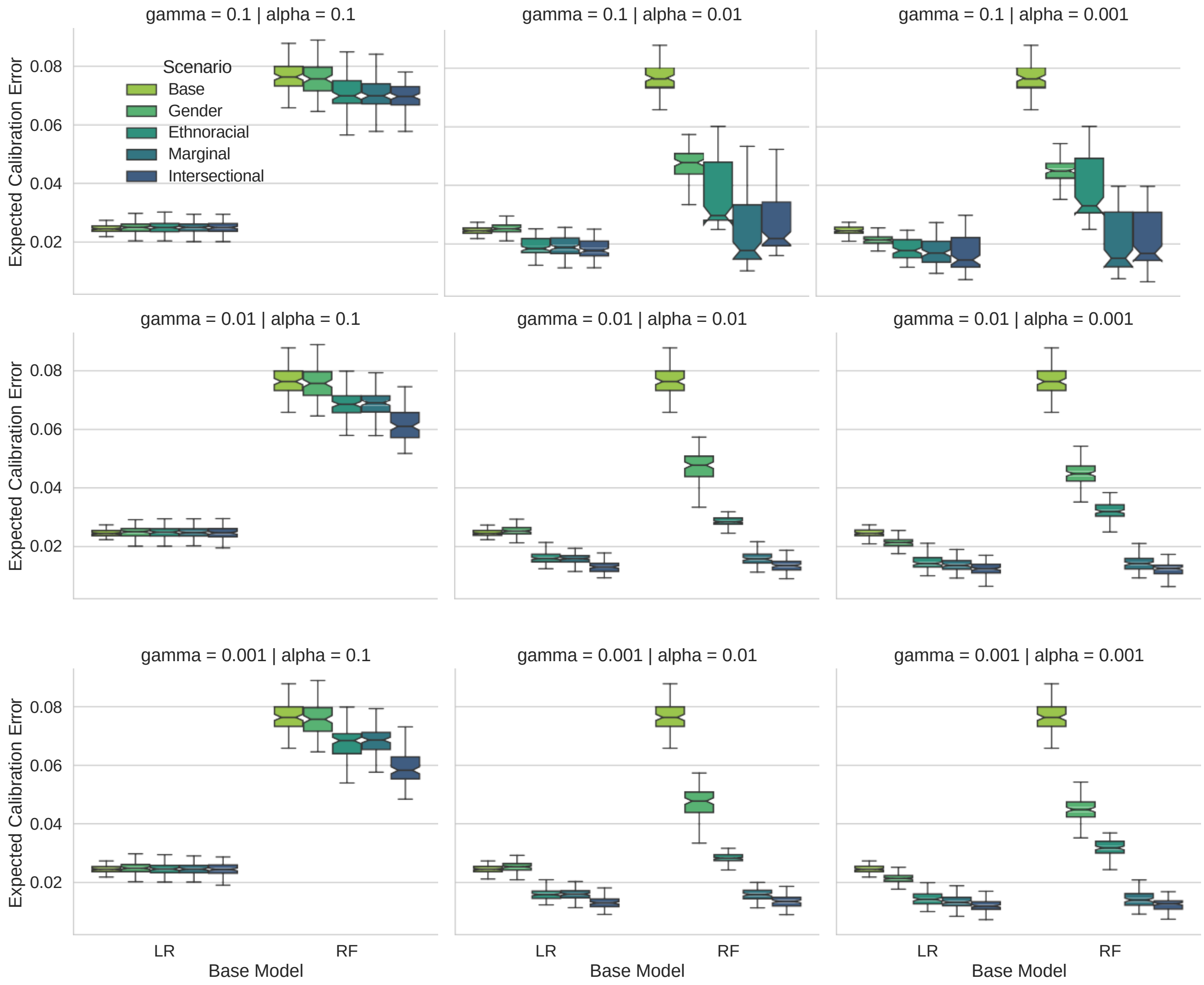

eFigure 2. Intersectional Group-Wise Expected Calibration Error on MIMIC-IV as a Function of γ (Row), α (Column), Base ML Model (X-Axis), and Optimization Scenario (Color). At high levels of α, the models remain unchanged, whereas at very low values of α and γ, performance on intersectional groups can suffer due to small sample sizes.

Multicalibration Boosting

Sensitivity Analysis. In Fig. S2, we visualize the expected calibration error of LR and RF models on MIMIC-IV as a function of base model, α, γ, and modeling scenario. At higher levels of γ, low-prevalence groups are excluded from fairness updating; hence, performance differences between scenarios tend to shrink. Relatedly, higher values of α loosen the threshold needed for multicalibration to perform an update, and so model performance tends to become similar between groups. Conversely, for very small values of α and γ, small groups have a larger impact on fairness optimization, meaning intersectional modeling matters more for achieving low ECE among intersectional groups. Overfitting can occur when α is too stringent, leading to degradation of performance on intersectional groups on test set: see top middle and right panel of Fig. S2, RF models.

Fig. S3 sheds light on the interaction between group prevalence, α and γ under multicalibration boosting.

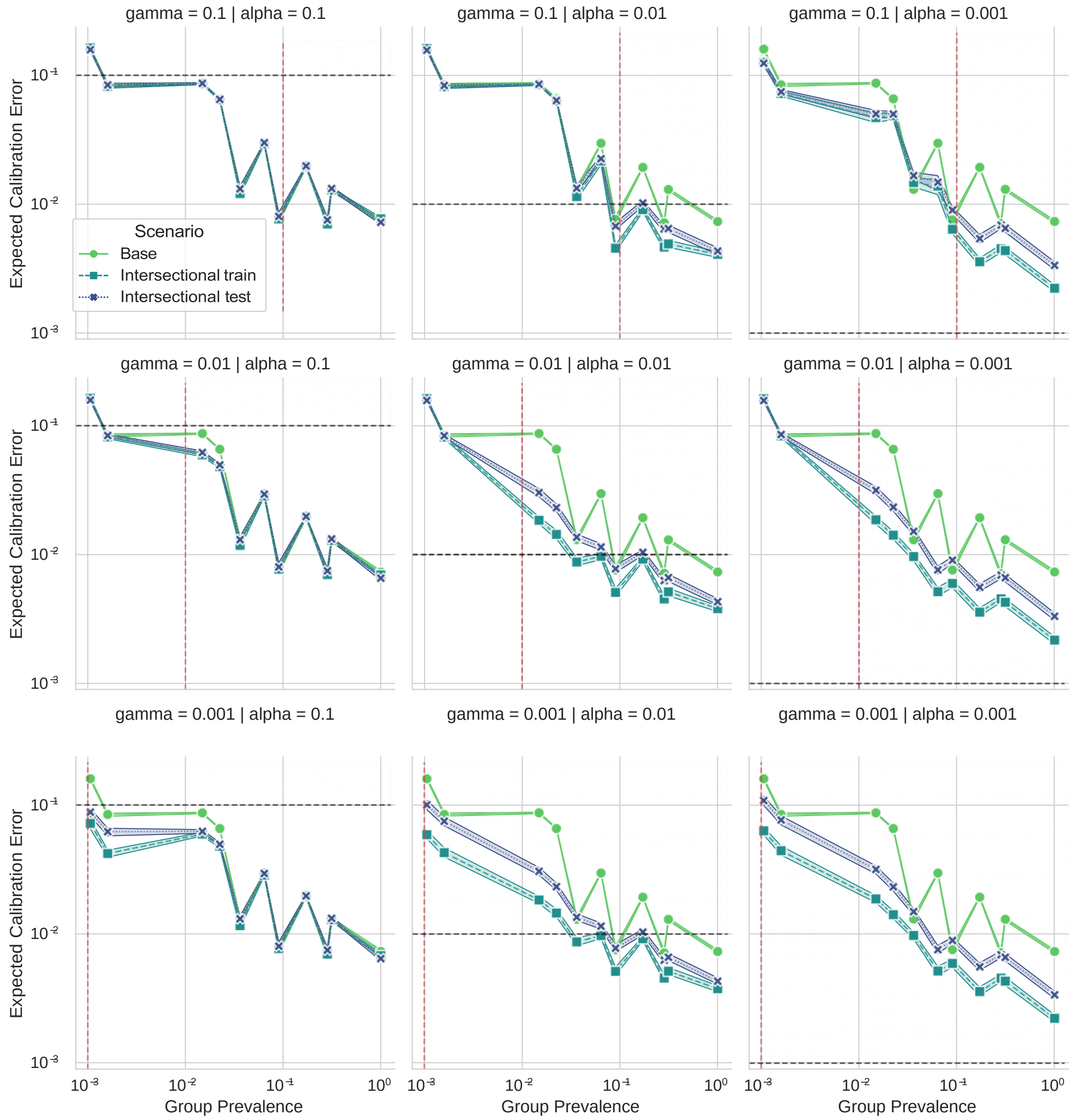

eFigure 3. Expected Calibration Error (ECE) as a Function of Group Prevalence for LR Models Trained on MIMIC-IV, Under Different Combinations of α and γ. The shaded area indicates the region of model performance that is subjected to optimization by either having an ECE higher than the threshold, α, or a group prevalence higher than the cutoff, γ.

© 2025 Lett E et al. *JAMA Network Open.*

Here we explicitly look at training and test set performance of the intersectional de-biasing approach relative to the baseline approach, illustrating how the constraints on calibration error (α) and minimimum group probability (γ) interplay with group prevalence (x-axis). In general, we observe that groups that are less prevalent in the data tend to have higher expected calibration error (ECE). Therefore, when α and γ are set high relative to model performance on adequately sized groups (e.g., $\alpha = \gamma = 0.1$, top left panel), no de-biasing occurs. Conversely, if γ and α is set very low, de-biasing occurs over all groups in the training data but this does not fully generalize to test data (bottom right panel).

Fairness-Oriented Multiobjective Optimization

Sensitivity Analysis. We varied several parameters during our experimentation with FOMO: 1) The choice of ML model (penalized logistic regression or random forests); 2) whether the definition of subgroup fairness incorporates the prior probability of the group as in other work [6]; 3) the type of meta-model used to estimate the sample weights used to train the base models. Regarding 1), we saw similar trends in results when working with linear models, as shown in Fig. S4. Regarding 2), we did not observe a difference in performance when incorporating prior probabilities of the groups; our results here do not incorporate these adjustments for group size. Regarding 3), we did not observe a difference in performance with variations of the meta-model. In our results, we use a standard linear formulation to map patient attributes to training sample weights; when using the intersectional fairness implementation, we extend the linear model with interaction terms between the scenario's protected features. Our observations suggest that whether or not the group probability was factored into the fairness definition, it had minimal discernible impact on the outcomes for both RF and LR models across both datasets.

Trade-off Visualization. Fig. S5 shows the set of models generated by FOMO as part of its optimization process, which characterizes the trade-off space (i.e. the Pareto frontier) between fairness and accuracy objectives.

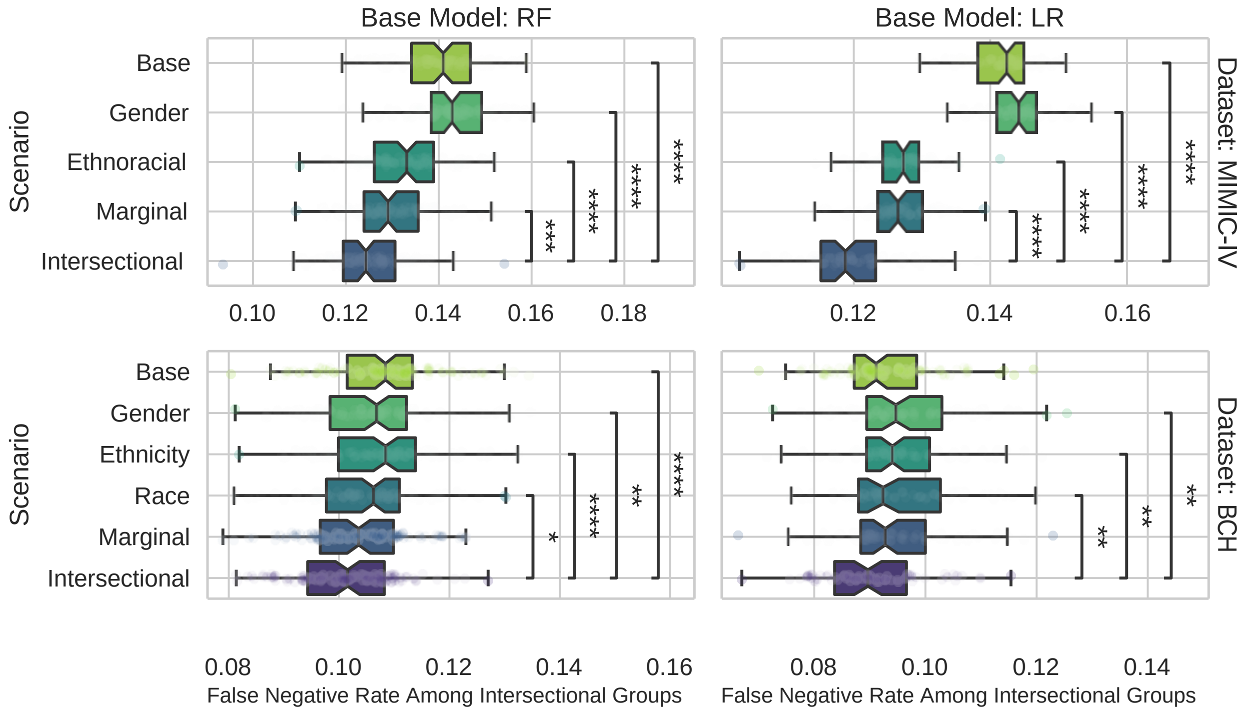

eFigure 4. False Negative Rates (FNR) Among Intersectional Groups Under Different Base Models (Left: Random Forests (RF), Right: Penalized Logistic Regression (LR)) and FOMO De-Biasing Scenarios (Y-Axis) for MIMIC-IV (Top) and BCH (Bottom). Statistical tests are two-sided Mann-Whitney-Wilcoxon tests with Holm-Bonferroni correction (*: 1e-2 < p <= 5e-2; **: 1e-3 < p <= 1e-2; ***: 1e-4 < p <= 1e-3; ****: p <= 1.0e-4).

© 2025 Lett E et al. *JAMA Network Open.*

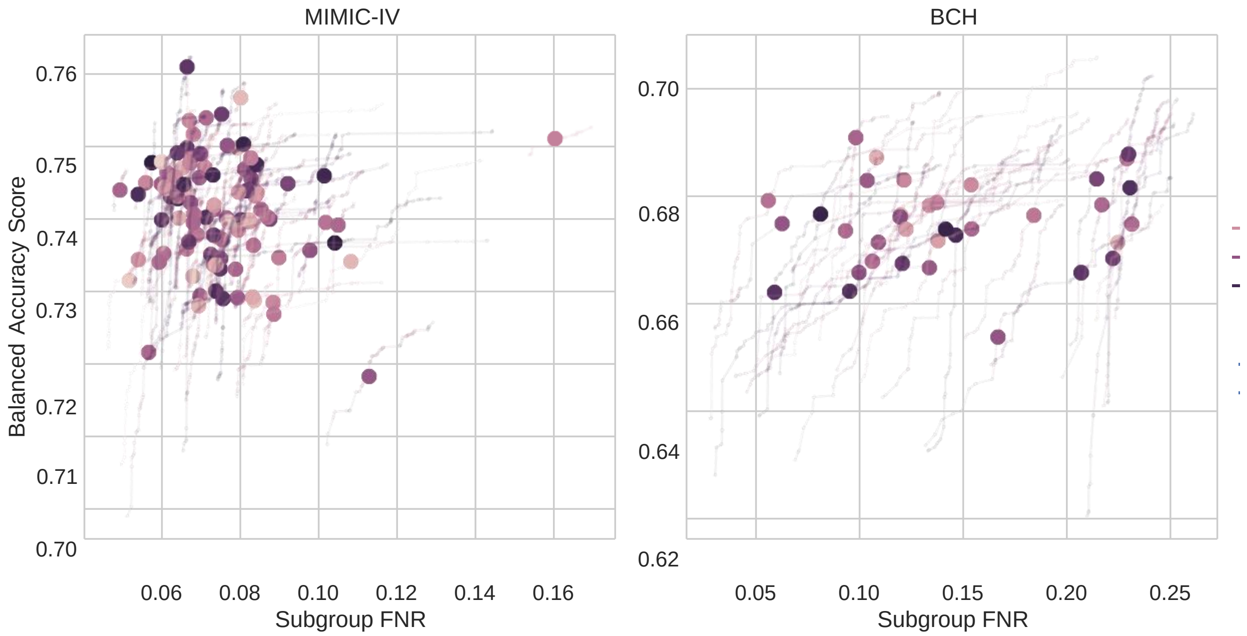

eFigure 5. Accuracy-Fairness Trade-Offs and Model Selection. FOMO optimizes a Pareto frontier of solutions simultaneously in order to characterize the trade-off between accuracy and fairness objectives. These final frontiers are shown for MIMIC-IV (left) and BCH (right), with each line representing one realization of the experiment. In order to choose a final model (marked by large circles for each run), a multi-criteria decision making method known as Pseudo-Weights is used [15]. This method chooses the model that maximizes a weighted sum of the objectives. For each candidate model, the weights of each objective depend on the normalized distance to the worst solution for that objective. FNR: false negative rate.

© 2025 Lett E et al. *JAMA Network Open.*

eReferences.

[1] Kamiran Faisal, Calders Toon. Data Preprocessing Techniques for Classification without Discrimination *Knowledge and Information Systems.* 2012;33:1–33.

[2] Rehman Ayaz Ur, Nadeem Anas, Malik Muhammad Zubair. Fair Feature Subset Selection Using Multiobjective Genetic Algorithm 2022.

[3] Zhang Brian Hu, Lemoine Blake, Mitchell Margaret. Mitigating Unwanted Biases with Adversarial Learning in *Proceedings of the 2018 AAAI/ACM Conference on AI, Ethics, and Society*AIES ’18(New York, NY, USA):335–340Association for Computing Machinery 2018.

[4] Keya Kamrun Naher, Islam Rashidul, Pan Shimei, Stockwell Ian, Foulds James. Equitable Allocation of Healthcare Resources with Fair Survival Models in *Proceedings of the 2021 SIAM International Conference on Data Mining (SDM)*:190–198SIAM 2021.

[5] Agarwal Alekh, Beygelzimer Alina, Dudik Miroslav, Langford John, Wallach Hanna. A Reductions Approach to Fair Classification in *International Conference on Machine Learning*:60–69 2018.

[6] Kearns Michael, Neel Seth, Roth Aaron, Wu Zhiwei Steven. Preventing Fairness Gerrymandering: Auditing and Learning for Subgroup Fairness *arXiv:1711.05144 [cs]*. 2018.

[7] La Cava William G.. Optimizing Fairness Tradeoffs in Machine Learning with Multiobjective Meta-Models in *Proceedings of the 2023 Genetic and Evolutionary Computation Conference (GECCO)*ACM.

[8] Hardt Moritz, Price Eric, Price Eric, Srebro Nati. Equality of Opportunity in Supervised Learning in *Advances in Neural Information Processing Systems 29* (Lee D. D., Sugiyama M., Luxburg U. V., Guyon I., Garnett R.. , eds.):3315–3323Curran Associates, Inc. 2016.

[9] Pleiss Geoff, Raghavan Manish, Wu Felix, Kleinberg Jon, Weinberger Kilian Q. On Fairness and Calibration in *Advances in Neural Information Processing Systems 30* (Guyon I., Luxburg U. V., Bengio S., et al. , eds.):5680–5689Curran Associates, Inc.

[10] Hebert-Johnson Ursula, Kim Michael, Reingold Omer, Rothblum Guy. Multicalibration: Calibration for the (Computationally-Identifiable) Masses in *Proceedings of the 35th International Conference on Machine Learning*:1939–1948PMLR.

[11] Kim Michael P., Ghorbani Amirata, Zou James. Multiaccuracy: Black-box Post-Processing for Fairness

© 2025 Lett E et al. *JAMA Network Open.*

in Classification in *Proceedings of the 2019 AAAI/ACM Conference on AI, Ethics, and Society*:247–254 2019.

[12] Tanabe Paula, Gimbel Rick, Yarnold Paul R., Kyriacou Demetrios N., Adams James G.. Reliability and Validity of Scores on The Emergency Severity Index Version 3 *Academic Emergency Medicine: Official Journal of the Society for Academic Emergency Medicine*. 2004;11:59–65.

[13] McLellan Mary C., Gauvreau Kimberlee, Connor Jean A.. Validation of the Cardiac Children's Hospital Early Warning Score: An Early Warning Scoring Tool to Prevent Cardiopulmonary Arrests in Children with Heart Disease ;9:194–202.

[14] La Cava William G., Lett Elle, Wan Guangya. Fair Admission Risk Prediction with Proportional Multicalibration in *Proceedings of the Conference on Health, Inference, and Learning*:350–378PMLR 2023.

[15] Deb Kalyanmoy. *Multi-Objective Optimization Using Evolutionary Algorithms*. John Wiley & Sons 2001.

[16] Pedregosa Fabian, Varoquaux Gaël, Gramfort Alexandre, et al. Scikit-Learn: Machine Learning in Python *Journal of Machine Learning Research.* 2011;12:2825–2830.

© 2025 Lett E et al. *JAMA Network Open.*
