# Supplementary material for: Intersectional and Marginal Debiasing in Prediction Models for Emergency Admissions
Source: JAMA Netw Open. 2025 May 29;8(5):e2512947. doi: 10.1001/jamanetworkopen.2025.12947 (PMC12123471; doi:10.1001/jamanetworkopen.2025.12947)
Supplement: Supplement 2. — Data Sharing Statement [file jamanetwopen-e2512947-s002.pdf]

Data Sharing Statement

Lett. Intersectional and Marginal Debiasing in Prediction Models for Emergency Admissions. *JAMA Netw Open*. Published May 29, 2025. doi:10.1001/jamanetworkopen.2025.12947

Data
Data available: No

Additional Information
Explanation for why data not available: Data Availability MIMIC-IV-ED is available from physionet.org/mimic-iv-ed. We provide full preprocessing code for the MIMIC-IV admissions dataset is available from the repository github.com/cavalab/mimic-iv-admissions. The BCH pediatric dataset is not publicly available under the terms of the BCH Institutional Review Board. Interested readers may contact the corresponding author for additional details. Code Availability The code for reproducing the experiments is available from github.com/cavalab/marginal-intersectional.
